# A Systems View of Emotion in Socio-political Context

**DOI:** 10.1007/s42761-021-00051-z

**Published:** 2021-07-07

**Authors:** Colin Wayne Leach, Fouad Bou Zeineddine

**Affiliations:** 1 Psychology & Africana Studies, Barnard College, Psychology, Faculty of Arts & Sciences, Data Science Institute, Institute for Research in African-American Studies, Columbia University, New York, NY USA; 2 Institut Für Psychologie, Universität Innsbruck, Innsbruck, Austria

**Keywords:** Emotion, Context, Social, Systems, Politics, Collective action

## Abstract

Most work to date in psychology and related sciences has examined simple, unidirectional causal processes of emotion affecting socio-political context or vice versa. In this classic, mechanistic view of science, each empirical observation stands on its own as a piece of some grander, not yet understandable, puzzle of nature. There have been repeated calls to eschew classic approaches in favor of systems meta-theory in psychology and related sciences. In this paper, we join these calls by arguing that systems meta-theory can better enable the study of emotions in socio-political contexts. We offer a brief primer on systems meta-theory, delineating three key beneficial features: multi-leveled, complex, and dynamic. Viewing emotion as a system of systems—within the person, their relationships (to others), and within the world (locally and globally)—enables fresh theory, method, and statistical analysis well suited to the study of emotion in a socio-political context.

Anyone can be angry—that is easy. But to be angry with the right person, to the right degree, at the right time, for the right purpose, and in the right way—that is not easy.

Nicomachean Ethics, Aristotle.

Most work to date has examined simple, unidirectional causal processes of emotion affecting socio-political context or vice versa (for reviews, see Parkinson et al., 2005; Tiedens & Leach, 2004). In this classic, mechanistic view of science, each empirical observation stands on its own as a piece of some grander, not yet understandable, puzzle of nature (for discussions, see Fodor, 1974; Leach, 2016; McGuire, 1983; Muthukrishna & Henrich, 2019). Thus, in some cases, one’s anger at believed injustice leads one to join others in protest, and in other cases, witnessing others protest against believed injustice leads one to anger. In the interests of getting on with the business of identifying and examining pieces of the puzzle, science defers an obvious question that might slow productivity: if we don’t know what the puzzle is (e.g., a Paris street scene), how can we fit the pieces together (i.e., distinguish a chapeau from a poodle)? This question has plagued (Western) science since its formal beginnings (Fodor, 1974; McGuire, 1983) and continues to do so (see Muthukrishna & Henrich, 2019).

An alternative to the classic view of science is to view phenomena as produced by systems within a system that organizes their relation in more complex ways than simple, unidirectional cause and effect. Such ideas go back at least as far as Aristotle. And recent moves to systems theory, method, and statistics have enriched the study of diverse phenomena including glacial melting, price changes, and disease spread (for reviews, see Eidelson, 1997; Vallacher et al., 2002; Wang & Grant, 2019). For instance, the modeling of weather as a complex system of systems (e.g., climate, ocean currents, wind, humidity) in the last decade has dramatically improved the forecasting of temperature, precipitation, and severe storms (see Bauer et al., 2015). Thus, in this paper, we offer a brief primer on systems meta-theory and argue that emotion in socio-political context is best understood as a system of systems, within the person (i.e., their goals, inclinations, concerns), within their relationships (to others), and within the world—local (i.e., family, work, school, neighborhood) and global (i.e., physical ecology, social structure, culture, societal values). Although the development of technical expertise in systems theory, method, and statistics in the field may take time, an embrace of systems meta-theory can already begin to inspire thinking and research that is more multi-leveled, causally complex, and dynamic.

## Systems Meta-theory

In psychology, and related social-behavioral sciences, repeated calls to eschew classic approaches in favor of systems meta-theory have been largely unsuccessful (for discussions, see Eidelson, 1997; Reilly et al., 2019; Thagard & Nerb, 2002; Vallacher et al., 2002). However, there is work consistent with systems meta-theory in grounded or embodied cognition (for reviews, see Niedenthal et al. 2009; Yeh & Barsalou, 2006), ecological (for recent discussions, see Meagher 2020; Read & Szokolszky, 2020) and socio-ecological (Uskul & Oishi, 2020) psychology, and situated social cognition (for a review, see Semin & Smith, 2013), for example. Systems meta-theory is also influencing the study of psychological concepts important to the study of emotion in socio-political contexts, such as power (Bou Zeineddine & Pratto, 2017; Gaski, 2020), influence (Vallacher et al., 2002), identity (e.g., Curtin et al., 2016; Hannah et al., 2020), and political personality (Reilly et al., 2019). There are three broad capacities in systems meta-theory that recommend it to the study of emotion in a socio-political context: multi-leveled, complex, and dynamic.

### Multi-leveled

According to the meta-theory there are multiple systems, at different levels of abstraction or of analysis, nested within a larger system. We may distinguish between macro-, meso-, and micro-levels (Greenaway et al., 2018) although finer distinctions are sometimes preferable (Bou Zeineddine & Pratto, 2017; Leach, 2010).

*Macro-level systems* include larger-scale contexts that operate through culture, structure, or institutions (Leach, 2010). These include the physical structure of objects, buildings, streets, neighborhoods (for a review, see Meagher, 2020), natural and artificial geographical boundaries (e.g., mountains, borders; see Rutherford et al., 2014), and political, economic, and other institutions (e.g., Bou Zeineddine & Pratto, 2017). Sociology and anthropology have typically been more focused on examining emotion as embedded in macro-level systems **(**for a recent review, see Bericat 2016).

*Meso-level systems* are typically considered as “bridges” between macro- and micro- systems because they are aspects of the immediate context (Leach, 2010). Such systems include relationships with smaller collectives such as couple, family, friends, neighbors, and co-workers (Greenaway et al., 2018). For example, the meso-level emotional climate or feeling norm of the collective can influence how individual members experience and express emotion via shared appraisal or feeling, social contagion, and dyadic synchronization (Parkinson et al., 2005; Smith & Mackie, 2015; Tiedens & Leach, 2004).

*Micro-level systems* typically refer to systems within the individual. These systems are the most frequently examined in work on emotion, especially in psychology and closely related disciplines like cognitive science or neuroscience (see Barrett, 2017; Sander et al., 2018). These include the perception, attention, memory, language, motor, nervous, and cardiovascular systems which are typically included in the common view of emotion as a syndrome or constellation of micro-level components (Beall & Tracy, 2017) that work together in a system or system-like process (for discussions, see Boiger & Mesquita, 2012; Bou Zeineddine & Leach, in press; Pessoa, 2019).

Recent advances in the ease and accessibility of multi-level statistical modeling have enabled more research to simultaneously examine meso-, macro-, or micro-level explanations of emotion in socio-political context and, in rare cases, to examine interactions between explanations at different levels. Systems meta-theory, however, goes beyond the fairly simple assumption that phenomena are produced by different systems that operate at different levels (Bou Zeineddine & Leach, in press). It assumes that these systems affect each other in *complex* and *dynamic* ways across levels (for discussions, see Eidelson, 1997; Vallacher et al., 2002; Wang & Grant, 2019).

### Complex

Systems meta-theory assumes that systems at multiple levels operate together in a complex (rather than simple, unidirectional, linear, mechanistic) way. This complexity is achieved because the component parts of each of the relevant systems, and thus the overarching system, is “self-organizing” (Eidelson, 1997; Wang & Grant, 2019). The parts combine to produce an indivisible whole that works to do what cannot be done by the parts on their own. Despite the fact that the systems may be nested in a hierarchical organization (e.g., from macro to micro, from earlier to later), no system is wholly dependent on or subservient to any other. Thus, each system is semi-autonomous. This means that a system of systems is redundant—there are multiple ways that components can combine to produce a phenomenon (see Wang & Grant, 2019). This is further reason to avoid simple, mechanistic *cause ➔ effect* theory, method, and statistical analysis.

Simple multi-level processes can be thought of as a (C) major chord in music, which combines (C, E, G) notes in a simple structure where it is readily apparent how the notes go together and why. In contrast, complex systems may be thought of as a non-obvious combination of a larger set of notes, that may even seem to be disparate or divisive, but out of which *emerges* a harmonic chord whose structure and logic are less apparent. If we were to exemplify complex systems in music, it would be something like John Coltrane’s modal jazz classic “A Love Supreme,” with its chromatic notes, reharmonization, key changes, and polyrhythms. Just as modal jazz liberated musicians to imagine and play music in new ways, systems meta-theory liberates scholars to theorize, study, and analyze emotion in socio-political contexts in new ways unconstrained by the mechanistic meta-theory of the classic view.

### Dynamic

Systems meta-theory argues that phenomena should be understood as dynamic (in time, across persons, and across contexts), rather than static (for reviews, see Eidelson, 1997; Vallacher et al., 2002; Wang & Grant, 2019). Indeed, “one never steps in the same river twice.” More technically, systems meta-theory presumes that phenomena exist in non-equilibrium states that may appear stable but are always changing (even if only to maintain the status quo). This change is often non-linear and even chaotic and must therefore be examined as highly probabilistic. Thus, systems meta-theory assumes heterogeneity in empirical observations, a good deal of which is random (McGuire, 1983; Muthukrishna & Henrich, 2019). Multi-level statistical analyses that specify mixed (fixed and random) effects and longitudinal modeling that examines heterogeneity of effects over time (e.g., latent growth curves) are consistent with the basics of systems thinking, especially if they include non-linearity in their analysis.

## Emotion Systems

At least since Sartre’s theory of emotion in the 1940s, scholars have labored to specify how emotion and socio-political context relate to one another in multi-leveled, complex, and dynamic ways (Leach, 2016; Leach & Tiedens, 2004; for reviews, Greenaway et al., 2018; Halperin, 2016; Parkinson et al., 2005; Smith & Mackie, 2015; Tiedens & Leach, 2004). For example, one strand of work ties micro-level processes of emotion to macro-level evolutionary forces such as sexual success, power and prestige, and protection within groups (see Al-Shawaf et al., 2015; Beall & Tracy, 2017; Sznycer, 2019).

Boiger and Mesquita’s (2012) contextualized view of emotion as rooted in interactions, relationships, and culture suggests that the social and psychological meaning most central to a particular emotion is variable. Indeed, they find that shame is more frequently about the loss of social image in Japan, whereas it is more about the personal defect in the USA. Cohen-Chen et al. (2020) showed that contexts such as intergroup conflict—where different parties have opposed values, goals, practices—alter the implications of emotion. Anger, for example, may be experienced as pleasant and empowering (rather than unpleasant), by those who view themselves as engaged in conflict to righteously resist oppression (see also Leach, 2016, 2020). Recent work on emotion regulation, emotion as an emergent phenomenon, and cognitive appraisal theory is more explicit in its alignment with systems meta-theory.

### Emotion Regulation

In the last few decades, work on individual control or “regulation” of emotion has advanced a system view of emotion (for a review, see Gross, 2015). This is likely because the regulation of emotion is obviously a dynamic process by which individuals aim to alter what, where, and for how long they experience an emotion (Kuppens & Verduyn, 2017). Work on emotion regulation is also suggestive of a system of systems view because it is increasingly clear that many different systems are involved. Recent research shows that emotional regulation involves multiple neural networks (e.g., Gruber & McDonald, 2012), with close associations to systems of attention (e.g., Wadlinger and Isaacowitz 2011), cognitive appraisal and learning (Webb et al., 2012), goals and expectations (Carver & Scheier, 2017), as well as evaluation of emotion-context match (Gross, 2015).

Recently, Ford et al. (2019) argued that people commonly use multiple regulation goals, strategies, or tactics in a “polyregulation” suggestive of complex multi-level dynamics. The influence of work on emotion regulation may be limited, however, by the stubborn presumption that emotion regulation is somehow a phenomenon distinct from the emotion itself (Gross, 2015; Kuppens & Verduyn, 2017). Until emotion regulation is viewed as part and parcel of (dynamic) emotion, the system of systems perspective prevalent in emotion regulation work may not serve as a basis for work on emotion in general (Kuppens & Verduyn, 2017).

### Emotion as an Emergent Phenomenon

At present, Barrett’s constructionist theory is among the most prominent examples of a system of the systems view of emotion (for reviews, see Barrett, 2017; Hoemann & Barrett, 2019). Central to her thinking is a disavowal of reductionist models of emotion, which conceptualize the phenomenon as built purely from fundamental, lower-order, biological processes in the brain and body. From this point of view, emotions are emergent phenomena produced by a complex system (of systems). This system is self-organized to make its best guess at a psychological state most appropriate to the situation at hand and its implications for the person’s preferences and goals. As a person’s emotion is their best guess at the meaning of a situation for them, this guess is adjusted as necessary when the guess is inappropriate or unhelpful to the situation or unacceptable to the person or important others (Hoemann & Barrett, 2019). In a related perspective, Thagard & Nerb (2002) call this phenomenon, emergent from cognitive and affective systems, an emotion gestalt.

### Cognitive Appraisal

In many ways, cognitive appraisal theory is the most longstanding and elaborated systems view of emotion, even if its system features are not always made explicit (for discussions, see Boiger & Mesquita, 2012; Leach, 2016, 2020; Pessoa, 2019; Scherer et al., 2001; Thagard & Nerb, 2012). For instance, cognitive appraisal theory has long viewed emotion as an emergent phenomenon produced by the interplay between numerous complex (neural, physiological, behavioral, cognitive, affective) systems (see Lazarus, 1991; Leach & Tiedens, 2004; Scherer et al., 2001).

**Fig. 1:**
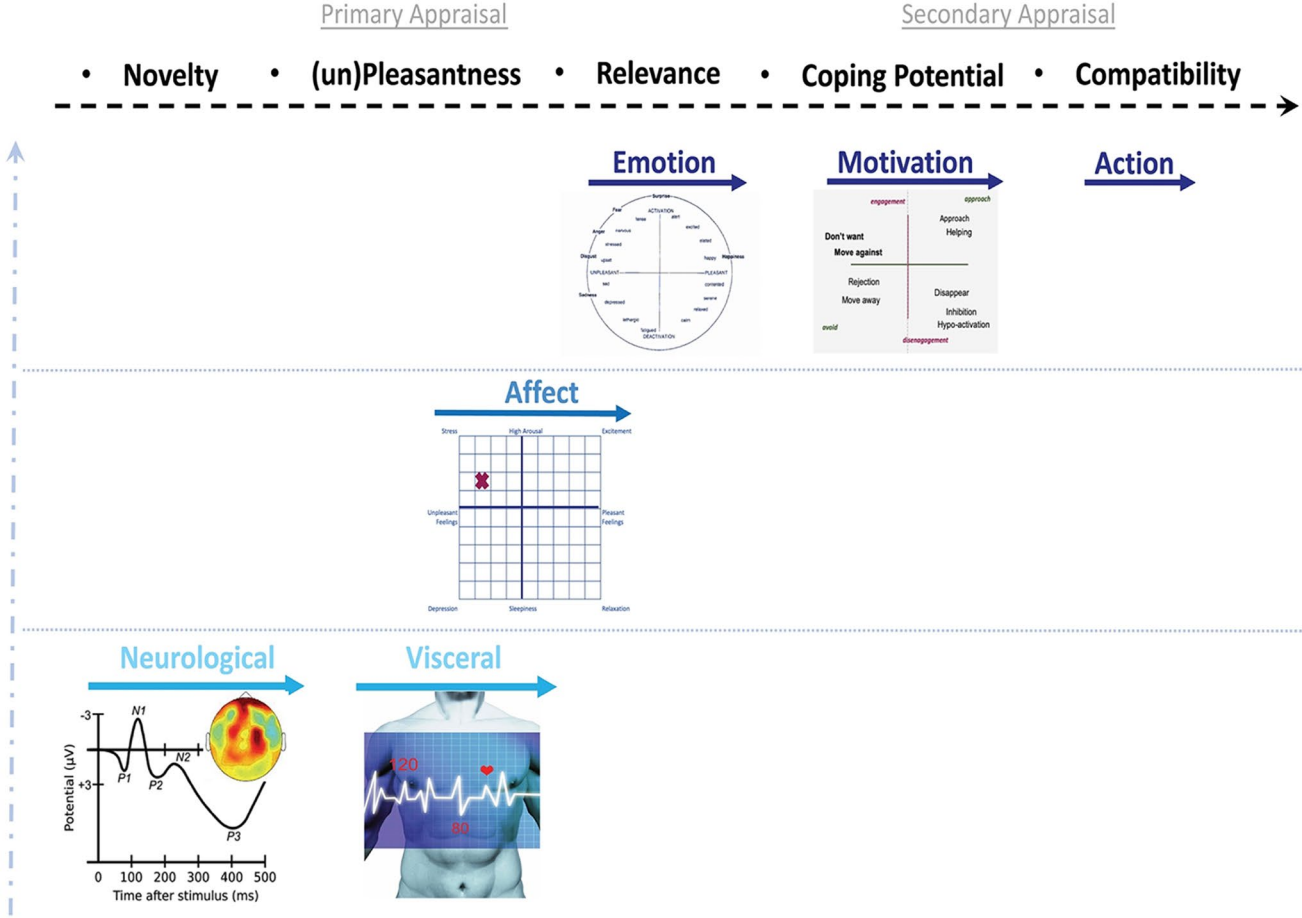
Temporal system of systems view of appraisal-emotion-motivation-action links

Recently, Sander et al. (2018) argued that cognitive appraisal proceeds in a logical, temporal order—novelty, (un)pleasantness, (motivational) relevance, coping potential, (social, value) compatibility (see the top of Fig. 1) that is constrained by the timescale of the systems that feed the appraisal (see Fig. 1). Despite the various timescales (from left to right) and system levels (from bottom to top) of cognitive appraisal theory, the systems meta-theory in which it is rooted argues against viewing the earlier systems (e.g., the neurological) as more foundational than any other. This is because emotion *emerges* from the complex dynamics of the whole system operating as one. In this sense, the directional arrows pointed up and right in Fig. 1 are no more than general guides about two particular parameters of the system (i.e., linear time and multiple systems). Any system can feed back into any other within the larger system. Although systems closer together in time and biological structure may most readily influence their neighbors, this is a probabilistic rather than deterministic feature of the complex structure and dynamics of the system (Reinka & Leach, 2018; Scherer et al., 2001).

## Emotion in Socio-political Context

**Fig. 2:**
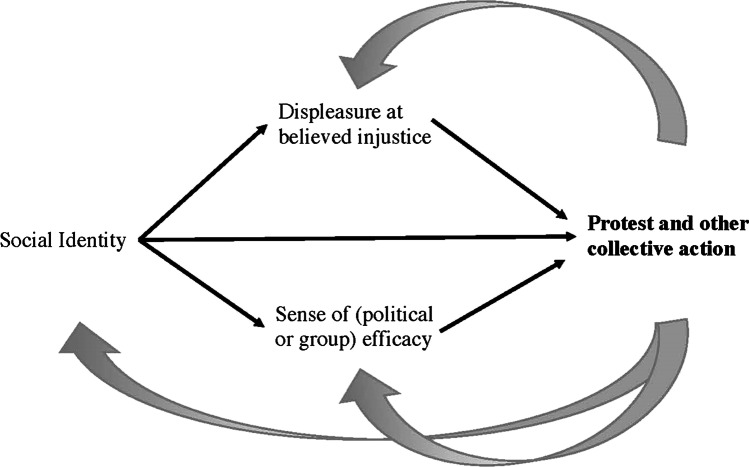
van Zomeren et al. (2008) Social Identity Model of Collective Action (SIMCA), with empirically observed “feedback loops” in grey

Research on the role of emotion in protest and other collective action against societal injustice has long aimed to incorporate macro- and meso-level aspects of socio-political context. van Zomeren et al. (2008) influential Social Identity Model of Collective Action (SIMCA) summarizes the key micro-level (i.e., psychological) explanations of protest as social identity, displeasure at believed injustice, and a sense of (political or group) efficacy to respond. As shown in Fig. 2, the model goes further in presuming that social identity as a member of a group that suffers societal injustice is a partial basis for displeasure at believed injustice and a sense of (political or group) efficacy.

A good deal of research shows, however, that social identity is also at times an effect of protest, as the failure or success of protest challenges or stimulates individual’s identification with their mistreated group (for reviews, see Louis et al., 2020; van Zomeren et al., 2012). The failure or success of protest can also affect displeasure at believed injustice (for a review, see van Zomeren et al., 2012) and a sense of efficacy to address it (for reviews, see Louis et al., 2020; van Zomeren et al., 2012). Thus, in contrast to the simple, unidirectional cause and effect process assumed by the SIMCA model (shown in black in Fig. 2), the individual motivation to protest is affected by feedback loops (shown in gray in Fig. 2). Evidence of such feedback loops is a sign that a phenomenon is likely better understood as multi-directional rather than unidirectional, dynamic rather than static, and complex rather than simple. In other words, the evidence of feedback loops in a process is a good sign that a systems approach is warranted.

**Fig. 3:**
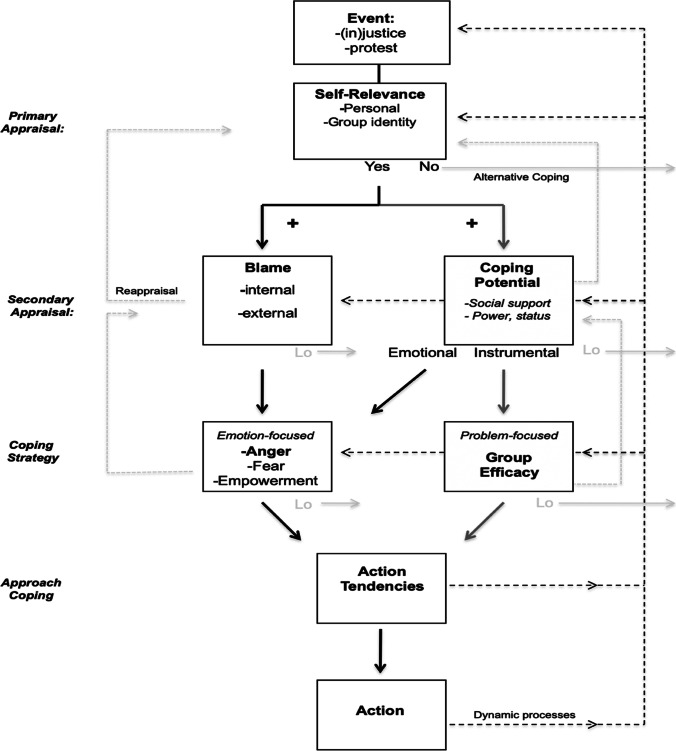
van Zomeren et al. (2012) dynamic dual pathway model of coping with societal disadvantage

One attempt at a systems view of individual motivation to protest is van Zomeren et al.’s (2012) dynamic model of coping with societal disadvantage, which has received far less attention than more classic approaches to much the same constructs. It is a system view because it is grounded in multi-system approaches to the micro-level processes of cognitive appraisal (see Scherer et al., 2001; Lazarus, 1991) and coping (see Lazarus, 1991), dynamically over time. The model allows “later” phenomena to influence “earlier” phenomena through feedback loops (shown in black dashed lines in Fig. 3), which include the process of reappraisal, whereby meaning is reassessed in light of new developments (shown in gray dashed lines in Fig. 3). A reappraisal is an important form of emotion regulation (for a review, see Gross, 2015).

The multi-level aspect of the model is shown in the inclusion of macro- and meso-level processes that are intimately connected to the micro-level processes of cognitive appraisal and coping. Rather than specifying individual belief in societal injustice as the only precipitating event for appraisal and coping, for instance, the model allows the macro-social phenomenon of a mass protest as a precipitating event. And, the appraisal of motivational relevance may be rooted in concern for one’s family, group, society, or planet. Rather than assuming that emotion- and problem-focused coping are solely micro-level individual processes, the model presumes that social support from like-minded others (at the meso-level) can be an important basis for emotion and a sense of (collective, political) efficacy.

A systems approach is also apparent in the model’s complexity, which allows alternatives to the focal process at each stage. A model that does not allow alternatives is too deterministic to be a (necessarily probabilistic) systems view. As shown in the solid gray lines of Fig. 2, the model specifies that alternative forms of coping are most probable when key appraisals are weak (for a review, see Leach, 2020). In addition, the model specifies that action tendencies do not necessarily translate to action, as concern for the ethical, legal, or existential implications of action like violent, disruptive, or otherwise radical protest may outweigh motivation to such a degree that the tendency to act is translated into more peaceful, cooperative, and accommodating protest or even inaction until the time is more ripe (see Becker & Tausch, 2015; van Zomeren et al., 2012).

Although less explicit in its reliance, another area of research that benefits from systems meta-theory is that on affect and emotion in the social-political context of the racialization of group membership, social relations, and attitudes toward policy and politics. Rooted in prior work on the neurological and physiological systems involved in attention to visual cues of race, dominance, and facial distress, Reinka and Leach (2018) used sequential cognitive appraisal theory to trace responses to images of police force against Black and White targets as well as images of Black Lives Matter protest. Consistent with their divergent ecological experiences, political interpretations, and familiarity with news of racialized police violence, Black and White Americans showed quite different patterns of appraisal, emotion, and motivation in response to the images across neurological, physiological, linguistic, and self-report indicators. It is central to systems thinking that our physical, social, and political ecologies shape what is novel, what is (un)pleasant, and what is relevant to us, even “deep down” at the level of our neurology, physiology, and other early systems of visual cognition and affect.

Given their study of individual group member’s reactions over time to multiple stimuli of different types, Reinka and Leach (2018) analyzed their data with mixed-effects models that could distinguish the effects of stimuli, stimuli type, individual, and individual’s group in ways that helped to identify the system likely in play for each effect. Additionally, at a macro-level, Riddle et al. (2020) examined some of these same images in conjunction with headlines in news coverage of a police killing of an unarmed Black teenager in Ferguson, Missouri. The differential use of images and words in Black-oriented vs. mainstream news suggested that the racialization of these information systems parallels that of the systems involved in the visual cognition and affect studied in Reinka and Leach (2018). As it is not always possible to examine systems at very distant levels in a single study, a multi-method project can rely on common concepts from a more general theory embedded within a systems meta-theory.

## Conclusion

Like Schill et al. (2019), we see great opportunities for systems meta-theory to enhance our understanding of human experience and behavior. One example of what is possible in the socio-political domain specifically is Vallacher et al.’s (2013) inter-disciplinary work on conflict. Their dynamical systems approach explains conflict as a system of systems that binds people in inertial patterns that are difficult to alter by simple changes to forces external to the system or by changes to superficial features of the system. They show how this conflict system is more easily altered by changes to the structure or dynamics of the system itself. This example shows why systems must be studied as systems, with tools to match from theory, method, and statistics (McGuire, 1983).

### Theory

To enact systems meta-theory, it must inspire the more specific and formalized thinking represented by theory (McGuire, 1983; Thagard and Nerb, 2012). Given its three key features, systems meta-theory requires theories that are more contingent, probabilistic, and holistic than theory rooted in a classic, mechanistic view (McGuire, 1983). Systems of emotion and context are necessarily “open” systems and thus, their dynamics are contingent to some degree on features that fluctuate. These could include macro-forces like political institutions or drought or micro-forces like mood or momentary salience. To theorize a system, one must incorporate these contingencies into theory in a formal way, rather than treating them as noise, post hoc moderators, or as unexamined “third variables” (McGuire, 1983). In other words, systems meta-theory demands that work on emotion in a socio-political context theorizes explicitly and formally how systems of emotion and context combine to produce phenomena of interest. Simple and sovereign pseudo-theories that assert mechanistic cause-effect processes, with no contingencies from higher- or lower-order systems—anger leads to aggression, shame leads to withdrawal, and guilt leads to restitution—can not be proper theories of a system (see Leach, 2016).

### Methods

As systems meta-theory is multi-leveled, it recommends multi-level methods (and statistics). And, as it is dynamic, it recommends the study of phenomena intensively over time (as well as over people and contexts). Given the complexity of systems, multiple methods are to be valued, as different methods used together provide a more complex combination of forms of empirical observation (McGuire, 1983). This is part of the reason that a systems approach to emotion in a socio-political context should be quite inclusive in its methods of research (see Bou Zeineddine & Leach, in press; Reilly et al., 2019). But, as systems meta-theory can embrace heterogeneous large-scale data from multiple levels, it is also a natural fit with newer “big data” and machine learning techniques (e.g., in analyses of the Twitter activity or digital footprint tracing), and with complex methods relying on design sciences, including experimental games and simulations (e.g., Reilly et al., 2019).

Indeed, systems research is often conducted using various forms of computational modeling to simulate the systems of interest. Computational modeling requires researchers to specify the key parameters of a system and then estimate how it operates over time, given specific starting assumptions, system structure, and system dynamics (for discussions, see Thagard and Nerb, 2012; Vallacher et al., 2002; Wang & Grant, 2019). Example methods include neural network models, complex adaptive systems, dynamic non-linear models, and agent-based models. For instance, one can imagine that current computational models of the spread of the virus that causes COVID-19 (in a city, region, country, hemisphere, world) can specify the role that fear of illness plays in the frequency of physical contact and thus spread. Key parameters might be (micro) intensity of individual fear and self-regulation skill to manage it, (meso) exposure to accurate information about virus spread and physical contacts aided by tracing of individual’s digital footprint, and (macro) levels of the virus in individual’s community and government measures against it. The model could be adjusted as these parameters change (e.g., as community infection rises or falls) or as a new parameter becomes important (e.g., a new more transmittable strain of the virus). In addition to requiring the formal (mathematical) specification of the key parameters of a system, computational modeling reinforces the system view that models are for continually estimating a range of probable trajectories that represent a best guess at any given moment.

### Statistics

Most work has yet to make full use of systems meta-theory, as very little of it uses methods or statistics to examine emotion operating as systems within a system (for discussion, and some exceptions, see Hollenstein, 2015; Thagard & Nerb, 2002). Given the principles of systems meta-theory, work must be more explicitly probabilistic than that rooted in a classic view of science (McGuire, 1983). As open systems cannot be predicted perfectly, systems research estimates the chances of a range of possible outcomes with stochastic methods. These explicitly incorporate randomness in the operation of systems. Through comparison to observations, models can be refined to improve prediction, especially if one can identify the key influences on system structure or dynamics. Thus, a key statistical approach in systems research is the comparison of model fit, power, and robustness (to perturbations in the system).

It is perhaps obvious that systems research on emotion and socio-political context cannot rely solely on frequentist, linear, cross-sectional statistics. A great deal of the data examined in systems work is non-linear time series. When data are non-linear, the common practice of simply correlating the indicators produced by different methods can be very misleading. For example, weak correlations between brain activity, blood pressure, and self-reported emotion, for instance, may say very little about the degree to which these three systems harmonize overtime to help produce emotion with a socio-political flavor (morally outraged anger) in the context of socio-political imagery or other stimuli (e.g., Reinka & Leach, 2018). Like notes and rhythm in modal jazz, the harmonics of these different systems are more complicated than can be examined with simple correlation. An important question at present is what the best statistical approach is for analyzing, as a unitary system, the complex, multi-method, and multi-level data representing the multiple systems involved in emotion in socio-politics. Intriguing possibilities include multi-level covariance structure models, continuous-time models, stable trait autoregressive trait and state models, parametric models, Gaussian graphical models, and spline functions.

Although it may take some time for scholars of emotion in socio-politics to embrace (and learn) the theory, methods, and statistics consistent with system meta-theory, the first step should be embracing the meta-theory, as it is the foundation that underlies the rest (McGuire, 1983). Doing so is not beyond the reach of any scholar in this area. Embracing system meta-theory should at least enable the re-imagining and reinterpretation of classical theory, methods, and statistics in system-like ways. A shared view of emotion in context as a system of systems should enable the diverse strands of work in this area to “speak a common language,” integrating the knowledge we have acquired thus far (see McGuire, 1983).

## References

[CR1] Al-Shawaf L, Conroy-Beam D, Asao K, Buss DM (2015). Human emotions: An evolutionary psychological perspective. Emotion Review.

[CR2] Barrett LF (2017). The theory of constructed emotion: An active inference account of interoception and categorization. Social Cognitive and Affective Neuroscience.

[CR3] Bauer P, Thorpe A, Brunet G (2015). The quiet revolution of numerical weather prediction. Nature.

[CR4] Beall, A.T., & Tracy, J.L. (2017). Emotivational psychology: How distinct emotions facilitate fundamental motives. *Social and Personality Psychology Compass,* e12303. 10.1111/spc3.12303

[CR5] Becker JC, Tausch N (2015). A dynamic model of engagement in normative and non-normative collective action: Psychological antecedents, consequences, and barriers. European Review of Social Psychology.

[CR6] Bericat E (2016). The sociology of emotions: Four decades of progress. Current Sociology.

[CR7] Boiger M, Mesquita B (2012). The construction of emotion in interactions, relationships, and cultures. Emotion Review.

[CR8] Bou Zeineddine, F. & Leach, C. W. (in press). Feeling and thought in collective action on social issues: Toward a systems perspective. *Social and Personality Psychology Compass*. 10.1111/spc3.1262

[CR9] BouZeineddine F, Pratto F (2017). The need for power and the power of need: An ecological approach for political psychology. Advances in Political Psychology.

[CR10] Carver CS, Scheier MF (2017). Self-regulatory functions supporting motivated action. Advances in Motivation Science.

[CR11] Cohen-Chen S, Pliskin R, Goldenberg A (2020). Feel good or do good? A valence-function framework for understanding emotions. Current Directions in Psychological Science.

[CR12] Curtin N, Kende A, Kende J (2016). Navigating multiple identities: The simultaneous influence of advantaged and disadvantaged identities on politicization and activism. Journal of Social Issues.

[CR13] Eidelson RJ (1997). Complex adaptive systems in the behavioral and social sciences. Review of General Psychology.

[CR14] Fodor, J. A. (1974). Special sciences (or: The disunity of science as a working hypothesis). *Synthese*, 97–115. Retrieved December 31, 2020, from www.jstor.org/stable/2011495810.1007/BF00485230

[CR15] Ford BQ, Gross JJ, Gruber J (2019). Broadening our field of view: The role of emotion polyregulation. Emotion Review.

[CR16] Gaski JF (2020). On contemporary misdefinition of power and the importance of definitional fidelity. Cogent Psychology.

[CR17] Greenaway KH, Kalokerinos EK, Williams LA (2018). Context is everything (in emotion research). Social and Personality Psychology Compass.

[CR18] Gross JJ (2015). Emotion regulation: Current status and future prospects. Psychological Inquiry.

[CR19] Gruber AJ, McDonald RJ (2012). Context, emotion, and the strategic pursuit of goals: Interactions among multiple brain systems controlling motivated behavior. Frontiers in Behavioral Neuroscience.

[CR20] Halperin E (2016). Emotions in conflict: Inhibitors and facilitators of peace making.

[CR21] Hannah ST, Thompson RL, Herbst KC (2020). Moral identity complexity: Situated morality within and across work and social roles. Journal of Management.

[CR22] Hoemann K, Barrett LF (2019). Concepts dissolve artificial boundaries in the study of emotion and cognition, uniting body, brain, and mind. Cognition and Emotion.

[CR23] Hollenstein T (2015). This time, it’s real: Affective flexibility, time scales, feedback loops, and the regulation of emotion. Emotion Review.

[CR24] Kuppens P, Verduyn P (2017). Emotion Dynamics. Current Opinion in Psychology.

[CR25] Lazarus RS (1991). Emotion and adaptation.

[CR26] Leach CW, Levine J, Hogg M (2010). Levels of analysis. Encyclopedia of group processes and intergroup relations.

[CR27] Leach CW (2016). The meta-theory of examining emotion in social relationships. Psychological Inquiry.

[CR28] Leach CW, Ray Vollhardt J (2020). Ways of coping with collective victimization. The social psychology of collective victimhood.

[CR29] Leach CW, Tiedens LZ, Tiedens LZ, Leach CW (2004). A world of emotion. The social life of emotions.

[CR30] Louis W, Thomas E, McGarty C, Lizzio-Wilson M, Amiot C, Moghaddam F (2020). The volatility of collective action: Theoretical analysis and empirical data. Political Psychology.

[CR31] McGuire WJ (1983). A contextualist theory of knowledge: Its implications for innovation and reform in psychological research. Advances in Experimental Social Psychology.

[CR32] Meagher BR (2020). Ecologizing social psychology: The physical environment as a necessary constituent of social processes. Personality and Social Psychology Review.

[CR33] Muthukrishna M, Henrich J (2019). A problem in theory. Nature Human Behaviour.

[CR34] Niedenthal PM, Winkielman P, Mondillon L, Vermeulen N (2009). Embodiment of emotion concepts. Journal of Personality and Social Psychology.

[CR35] Parkinson B, Fischer AH, Manstead ASR (2005). Emotion in social relations: Cultural, group, and interpersonal processes.

[CR36] Pessoa L (2019). Embracing integration and complexity: placing emotion within a science of brain and behaviour. Cognition and Emotion.

[CR37] Read, C., & Szokolszky, A. (2020). Ecological psychology and enactivism: Perceptually-guided action vs. sensation-based enaction. *Frontiers in Psychology, 11*. 10.3389/fpsyg.2020.01270 10.3389/fpsyg.2020.01270 PMC7381233 32765330

[CR38] Reilly A, Rooy DV, Angus S (2019). A complex adaptive systems approach to the relationship between personality and social division. Systems Research and Behavioral Science.

[CR39] Reinka MA, Leach CW (2018). Racialized images: Tracing appraisals of police force and protest. Journal of Personality and Social Psychology.

[CR40] Riddle T, Turetsky K, Bottesini J, Leach CW (2020). “What’s going on” in Ferguson? Online News of Protest at the Police Killing of Michael Brown. Group Processes and Intergroup Relations.

[CR41] Rutherford, A., Harmon, D., Werfel, J., Gard-Murray, A. S., Bar-Yam, S., Gros, A., ... & Bar-Yam, Y. (2014). Good fences: The importance of setting boundaries for peaceful coexistence. *PLoS One*, *9*(5), e95660. 10.1371/journal.pone.0095660 PMC4029557 24847861

[CR42] Sander D, Grandjean D, Scherer KR (2018). An appraisal-driven componential approach to the emotional brain. Emotion Review.

[CR43] Scherer KR, Schorr A, Johnstone T (2001). Appraisal processes in emotion: Theory, methods, research.

[CR44] Schill, C., Anderies, J. M., Lindahl, T., Folke, C., Polasky, S., Cárdenas, J. C., ... & Schlüter, M. (2019). A more dynamic understanding of human behaviour for the Anthropocene. *Nature Sustainability*, 1-8. 10.1038/s41893-019-0419-7

[CR45] Semin GR, Smith ER (2013). Socially situated cognition in perspective. Social Cognition.

[CR46] Smith ER, Mackie DM (2015). Dynamics of group-based emotions: Insights from intergroup emotions theory. Emotion Review.

[CR47] Sznycer D (2019). Forms and functions of the self-conscious emotions. Trends in Cognitive Sciences.

[CR48] Thagard P, Nerb J (2002). Emotional gestalts: Appraisal, change, and the dynamics of affect. Personality and Social Psychology Review.

[CR49] Tiedens, L. Z., & Leach, C. W. (2004) (Eds.), *The social life of emotions*. Cambridge University Press. 10.1017/CBO9780511819568

[CR50] Uskul AK, Oishi S (2020). Socio-ecological psychology [special issue]. Current Opinion in Psychology.

[CR51] Vallacher RR, Coleman P, Nowak A, Bui-Wrzosinska L, Liebovitch LS, Kugler K, Bartoli A (2013). Attracted to conflict: Dynamic foundations of destructive social relations.

[CR52] Vallacher RR, Read SJ, Nowak A (2002). The dynamical perspective in social psychology. Personality and Social Psychology Review.

[CR53] van Zomeren M, Leach CW, Spears R (2012). Protesters as “passionate economists” a dynamic dual pathway model of approach coping with collective disadvantage. Personality and Social Psychology Review.

[CR54] van Zomeren M, Postmes T, Spears R (2008). Toward an integrative social identity model of collective action: A quantitative research synthesis of three socio-psychological perspectives. Psychological Bulletin.

[CR55] Wadlinger HA, Isaacowitz DM (2011). Fixing our focus: Training attention to regulate emotion. Personality and Social Psychology Review.

[CR56] Wang H-H, Grant WE (2019). Ecological modeling: An introduction to the art and science of modeling ecological systems.

[CR57] Webb TL, Miles E, Sheeran P (2012). Dealing with feeling: a meta-analysis of the effectiveness of strategies derived from the process model of emotion regulation. Psychological Bulletin.

[CR58] Yeh, W., & Barsalou, L. W. (2006). The situated nature of concepts. *The American Journal of Psychology*, 349-384. 10.2307/20445349 17061691

